# Identification of Emotional Expression With Cancer Survivors: Validation of Linguistic Inquiry and Word Count

**DOI:** 10.2196/18246

**Published:** 2020-10-30

**Authors:** Michelle McDonnell, Jason Edward Owen, Erin O'Carroll Bantum

**Affiliations:** 1 Veteran's Affairs Loma Linda Healthcare System Loma Linda, CA United States; 2 US Department of Veterans Affairs, National Center for PTSD, VA Palo Alto Health Care System Palo Alto, CA United States; 3 Cancer Prevention in the Pacific University of Hawaii Cancer Center Honolulu, HI United States

**Keywords:** linguistic analysis, emotion, validation

## Abstract

**Background:**

Given the high volume of text-based communication such as email, Facebook, Twitter, and additional web-based and mobile apps, there are unique opportunities to use text to better understand underlying psychological constructs such as emotion. Emotion recognition in text is critical to commercial enterprises (eg, understanding the valence of customer reviews) and to current and emerging clinical applications (eg, as markers of clinical progress and risk of suicide), and the Linguistic Inquiry and Word Count (LIWC) is a commonly used program.

**Objective:**

Given the wide use of this program, the purpose of this study is to update previous validation results with two newer versions of LIWC.

**Methods:**

Tests of proportions were conducted using the total number of emotion words identified by human coders for each emotional category as the reference group. In addition to tests of proportions, we calculated F scores to evaluate the accuracy of LIWC 2001, LIWC 2007, and LIWC 2015.

**Results:**

Results indicate that LIWC 2001, LIWC 2007, and LIWC 2015 each demonstrate good sensitivity for identifying emotional expression, whereas LIWC 2007 and LIWC 2015 were significantly more sensitive than LIWC 2001 for identifying emotional expression and positive emotion; however, more recent versions of LIWC were also significantly more likely to overidentify emotional content than LIWC 2001. LIWC 2001 demonstrated significantly better precision (F score) for identifying overall emotion, negative emotion, and anxiety compared with LIWC 2007 and LIWC 2015.

**Conclusions:**

Taken together, these results suggest that LIWC 2001 most accurately reflects the emotional identification of human coders.

## Introduction

Recent studies have provided evidence that emotions can be effectively identified in written text [1-4]. Written emotions have been identified as significantly different from characteristically nonemotional writing, such as academic tasks [4], and more importantly, they can be correctly identified by readers [3]. As the use of web-based interventions, mobile app, social media, other text communication (eg, emails and text messages), and web-based communication (eg, Zoom and Skype) increases, the need for validated tests that can rapidly analyze text data has become exceptionally important. A large proportion of emotion-based text data are currently analyzed by human coders [5,6] as they add predictive power above and beyond that of computer analyses for the identification of positive emotions [7] as well as symptoms of depression [8]. Although computer analysis has become increasingly more efficient in evaluating written text, it lacks the nuance and accuracy provided by human coders [9]. Although qualitative analysis provides the most complete method for characterizing text-based communications [10], the cost, time requirements, and subjectivity of manual coding make these methods prohibitively difficult for many applications. Computerized text analysis programs may be able to ameliorate these limitations. Unfortunately, many computerized text analysis programs are not validated for the identification of emotional expression and still have time-consuming data preparation to ensure that the text is clear of all typographical errors [9].

The Linguistic Inquiry and Word Count (LIWC) [11] is a text analysis program that calculates the degree to which various categories of words are used in text. Bantum and Owen [12] previously evaluated the validity of the LIWC 2001, demonstrating that LIWC 2001 had good sensitivity and specificity for identifying emotion; however, the positive predictive value (PPV), or precision of emotional identification, was poor. Additional work by our team with the creation of a machine learning program [13] demonstrated that a machine learning approach was not necessarily more predictive than LIWC. Since our previous validation study, Pennebaker et al [14] have released 2 updates: LIWC 2007 and LIWC 2015, both of which have removed categories found to have poor emotional identification (eg, optimism) and increased the dictionary size of the program. These updates have greatly altered the dictionary for this program, including new categories of drive, analytical thinking, emotional tones, time orientation, and relativity and new subcategories of gender references and netspeak. In terms of emotional content, the largest change involves the inclusion of *netspeak* to quantify emotional expression (eg, “:)” would be categorized as a positive emotion word).

It is essential to validate LIWC 2007 and LIWC 2015 due to the widespread use of LIWC in research, clinical treatment, and commercial applications. LIWC 2001 and 2015 successfully identified individuals at risk for suicide as they had an increased presence of first-person singular self-references and negative emotional expression as they approached their suicide [1,2,15]. Sonnenschein et al [16] indicated LIWC 2007 noted that depressed individuals produced more words related to the sadness subcategory when compared with anxious individuals. Overall, LIWC has been determined to be able to accurately identify language differences between people who are diagnosed with attention deficit hyperactivity disorder, anxiety, bipolar, borderline, depression, eating disorders, obsessive-compulsive disorder, schizophrenia, and seasonal affective disorder [17,18]. LIWC 2007 has been used to evaluate whether a clinician’s distancing language increases suicide rates among veterans [19], assess what men and women value in romantic relationships [20], determine how social media predicts the outcomes of presidential elections [21], and evaluate language style and its ability to determine relationship initiation and stability [22].

Similar to LIWC 2001, the original evaluation of the validity of LIWC 2007 and LIWC 2015 was based on the results of a series of correlations between judges’ ratings and LIWC scores. Bantum and Owen [12] found that LIWC have low predictive value and overidentification of emotions, despite significant, though modest, correlations between judges’ ratings and LIWC scores [12]. Given these shortcomings and the need to independently validate LIWC 2007 and LIWC 2015 for emotion recognition in text [23,24], we sought to determine whether LIWC 2007 and LIWC 2015 are valid for emotion recognition in text and whether they improve upon the known limitations of LIWC 2001. The first aim of this study was to assess the accuracy of LIWC 2007 and LIWC 2015 for the detection of emotional expression using tests of specificity, sensitivity, PPV, and negative predictive value (NPV). The second aim was to evaluate potential differences between LIWC 2001, LIWC 2007, and LIWC 2015 for emotional identification. It is hypothesized that LIWC 2015 will be more efficacious in emotional identification than LIWC 2007 and LIWC 2001 because of the significant alterations in the most recent dictionary, recent regard for some contextual information in written text, and continued improvement in word selection through the construction of the product.

## Methods

### Participants

The participants were recruited from a hematology/oncology outpatient clinic at a large medical center in the southeastern United States. The participants included 49 women with stage 1 or stage 2 breast cancer and 14 women with stage 3 or stage 4 breast cancer. Participants were not excluded from participation based on the time elapsed since their diagnosis or medical treatment. The women participated in a randomized 12-week clinical trial of an internet-based support group. Additional information regarding the sample has been previously reported [25]. The textual data of the 63 participants were analyzed for this particular study. The participants had a mean age of 49.8 years (SD 11.0), the majority were college educated (mean 15.4 years, SD 2.4), and they were largely of White descent (57/63, 93%).

### Procedures

All participants completed a baseline assessment once they agreed to participate in the study, before being given access to the web-based support group. Once the participants were given access to the web-based support group, they were encouraged to communicate with one another through a discussion board regarding general topics and a series of interactive coping-skills training. The textual data were stored in an individual data file for each participant. Further information regarding the experimental procedures for these participants has been previously reported [12,26]. The study data set is identical to that used in the original validation study of LIWC 2001.

### Rater Coding of Emotional Expression

This particular study utilized human-coded ratings of emotion generated in a previous analysis of these linguistic data [12]. To briefly describe how these codes were generated, Bantum and Owen [12] had a well-defined set of rater coding rules for the human coders to follow. Each coder independently identified all words containing emotional expression. If the coders determined that emotional expression was present in a given sentence (as taken into consideration within the context of the text), then the word that most acutely captured the emotion was placed into the best fitting subcategory, based on categories specified by LIWC 2001. Eight potential categories were used: “positive feelings,” “optimism,” “anxiety,” “anger,” “sadness,” “other positive emotion,” “other negative emotion,” or “not emotion.” Coders were asked to differentiate between what was likely to be a physical pain and emotional pain or experience (eg, “the impact of chemotherapy on me physically was physically exhausting”). This process took quite a lot of training and judgment, as the use of emotion can be ambiguous, especially solely in text. Any discrepancies found between coders were discussed among the researchers, and final codes were established by consensus. The interrater reliability between the two trained coders was excellent (κ=0.80). Two additional raters were trained on the coding process and then reviewed 33% of the text. The interrater reliability was evaluated between the two additional raters and was established to have substantial agreement between raters (κ=0.69).

### Materials

#### LIWC 2001

LIWC 2001 is a computational text analysis method that processes text-based data on a word-by-word basis. LIWC compares each word of text with a library of 5 categories (ie, linguistic dimensions, psychological processes, relativity, personal concerns, and experimental dimensions) of words to identify whether each specific word from the source data set matches any of the words or word fragments found in the LIWC library. With regard to emotion, words are compared with each of the 3 categories of emotion (eg, emotional expression, positive emotion, and negative emotion). For each word that is established as a match to a word or fragment in a LIWC emotion dictionary, the program iterates a count of all emotion words identified in that particular emotion dictionary (eg, positive emotion). LIWC uses the results of the word count to establish a percentage of total words in the text that contain emotion words or a specific type of emotion. After the word has been identified as positive or negative, it is then placed into a specific category, such as positive feelings or optimism in the positive category and sadness, anger, or anxiety in the negative category. In some instances, words may be identified as expressing emotion and categorized as positive or negative, but LIWC is unable to resolve a more specific emotion category (eg, anxiety vs sadness), which may cause it to identify a word as belonging to multiple emotion dictionaries.

#### LIWC 2007

Each participant’s text information was also analyzed using LIWC 2007 (n=63) [14]. LIWC 2007 has a similar structure to that of LIWC 2001 in that it is a computational text analysis program that evaluates each item on a word-by-word basis. Furthermore, LIWC 2007 also provides a percentage of total words that are represented by emotion.

Pennebaker et al [14] developed LIWC 2007 to specifically address a number of key limitations in LIWC 2001, such as a limited dictionary, uncommonly used word categories, and a lack of function words (eg, conjunctions, adverbs, quantifiers, auxiliary verbs, and impersonal pronouns). The creators of LIWC 2007 removed the following word categories found in LIWC 2001 because they had poor base rates: optimism, positive feelings, communication verbs, metaphysical, sleeping, grooming, and school. The new dictionary for LIWC 2007 was altered to provide more accurate word categories by omitting those categories with insufficient validity and adding a number of categories to represent function words as well as including previously experimental categories into the program (eg, swear words, nonfluencies, and fillers). Additionally, researchers increased the dictionary count from 2300 words and word stems to 4500 words so that it may better represent emotional expression and other key psychological constructs. There were a number of words defined as emotion in the LIWC 2007 dictionary that were previously categorized as nonemotion (eg, confident, champ, and resolve). In addition to a reclassification of preexisting words, LIWC 2007 added emotional words that were not originally included in the LIWC 2001 dictionary (eg, grace, jaded, joke, openness, and rancid) and removed some emotional words that were in the LIWC 2001 dictionary (eg, sensitive). Finally, the LIWC 2007 dictionary classified the roots of words as emotion (eg, stammer) that may be perceived as nonemotion by human coders in an extended form (eg, stammered and stammering).

#### LIWC 2015

Each participant’s text information was also analyzed using LIWC 2015 (n=63) [27]. LIWC 2015 has a similar structure to the previous versions in that it evaluates textual data on a word-by-word basis, looking for specific target words within the appropriate word category scales. As words are identified as target words, they are classified into one or more categories or subcategories, which are then arranged hierarchically. Specifically, the word “cried” may be placed in the categories of sadness, negative emotion, overall affect, verbs, and past focus. Furthermore, LIWC 2015 provides a percentage of total words that are represented by emotion, and various structural composition elements (eg, sentence punctuation) are also incremented.

Pennebaker et al [27] created an entirely new dictionary and software system, rather than simply updating the previous versions of LIWC. This resulted in an increase in the dictionary size, totaling nearly 6400 words, word stems, and select emoticons. In addition to an increase in dictionary size, LIWC 2015 added 9 word categories tapping into psychological constructs (eg, affect and cognition) and 5 informal language categories (assents, fillers, swear words, and netspeak). There was the removal of one personal concern category (eg, work, home, and leisure activities) and one standard linguistic category (eg, percentage of words in the text that are pronouns, articles, and adjectives). Pennebaker et al [27] also reclassified how the word “like” is categorized in an attempt to reduce the risk of false positives that may occur in utterances, comparatives, or prepositions. Specifically, in previous versions of LIWC, the word “like” was categorized as indicative of emotion, particularly positive emotion. In LIWC 2015, the word “like” only qualifies as an emotion word when attributed to a person or an action, such as “I like,” “they like,” and “will like.” Pennebaker et al [27] added a category of *Netspeak* to assess for words and communication styles (eg, btw, lol, and thx) common on social media platforms (eg, Facebook, Snapchat, and instant messaging).

### Data Preparation

Each time the individuals participated in the web-based support group, their textual data were saved in person-specific files. The files were then combined into a single spreadsheet per participant so that each word was considered a subject. The text files from the human rater coding of emotional expression were created and combined with the results from the LIWC 2001, LIWC 2007, and LIWC 2015 data analysis. Each instance of emotion was counted as one point, and the frequency of a given emotion was divided by total words for that participant, resulting in a percentage of a given emotion for each participant. This was true for LIWC 2001, LIWC 2007, and LIWC 2015.

### Data Analytic Plan

This study contains a total of 165,754 words consisting of 278 single-spaced, 12-point font pages. Each word is considered as a single variable. An analysis of power was conducted using G*Power 3 [28] and indicated that with an effect size of 0.5, alpha level of .01, and a sample size of 165,754, the power to detect between-method differences in greater than 0.80. To assess the differences between LIWC 2001, LIWC 2007, and LIWC 2015 with regard to the accuracy of emotional identification, we calculated tests of proportions. We calculated the sensitivity, specificity, PPV, and NPV to identify the proportion of words that were similarly identified by human coders for LIWC 2001, LIWC 2007, and LIWC 2015. Subsequently, we utilized the overall proportions for each emotional category and conducted the test of proportions using the total number of emotion words identified by human coders for each emotional category as the reference group. To control for the number of tests, we calculated a Bonferroni correction for the *P* value to provide more stringent criteria for meeting sensitivity, which was calculated at *P*=.008. In addition to tests of proportions, we calculated F scores to evaluate the accuracy of LIWC 2001, LIWC 2007, and LIWC 2015. Accuracy was assessed by considering the harmonic mean of precision, or the fraction of retrieved items that are relevant, and recall, or the fraction of retrieved items that are relevant and successfully retrieved, of each program. This is a way of balancing the various measures (sensitivity and PPV) that we have evaluated. The F score was calculated by multiplying 2 with the results of PPV (precision) multiplied by sensitivity (recall) divided by PPV (precision) added to sensitivity (recall).

## Results

### Overview

The average percentage of words identified by LIWC 2001, LIWC 2007, LIWC 2015, and human coders as emotion, positive emotion, and negative emotion as well as specific subcategories of anxiety, anger, and sadness ranged from 0.1% to 4.1% of total words (Table 1). Pearson correlations were conducted to evaluate the strength of the relationship between each version of LIWC as well as human coders (Tables 2 and 3). The results revealed significant positive relationships across each version of LIWC, particularly between LIWC 2007 and LIWC 2015 (affect, *r*=0.95, *P*<.01; positive, *r*=0.95, *P*<.01; negative, *r*=0.97, *P*<.01; anxiety, *r*=0.97, *P*<.01; anger, *r*=0.91, *P*<.01; and sad, *r*=0.90, *P*<.01). With regard to the relationship between LIWC and human coders, the largest positive relationships occurred between LIWC 2001 (positive, *r*=0.47, *P*<.01; negative emotions, *r*=0.58, *P*<.01; and anxiety, *r*=0.74, *P*<.01) and LIWC 2015 (affect, *r*=0.55, *P*<.01; anger, *r*=0.51, *P*<.01; and sad, *r*=0.57, *P*<.01).

**Table 1:** Linguistic Inquiry and Word Count 2001, 2007, 2015, and human coder average of words identified in each emotional category and subcategory.

Type of emotion	LIWC^a^ 2001, % (SD)	LIWC 2007, % (SD)	LIWC 2015, % (SD)	Human coders, % (SD)
Total affect	4.8 (0.214)	6.1 (0.239)	6.1 (0.239)	1.8 (0.134)
Positive emotion	3.2 (0.175)	4.1 (0.198)	4.1 (0.198)	0.9 (0.096)
Negative emotion	1.6 (0.125)	1.9 (0.137)	1.9 (0.138)	0.9 (0.094)
Anxiety	0.5 (0.067)	0.6 (0.079)	0.6 (0.077)	0.3 (0.058)
Anger	0.2 (0.046)	0.2 (0.050)	0.2 (0.046)	0.1 (0.034)
Sadness	0.3 (0.059)	0.4 (0.063)	0.2 (0.066)	0.2 (0.044)

^a^LIWC: Linguistic Inquiry and Word Count.

**Table 2:** Correlations between Linguistic Inquiry and Word Count 2001, 2007, and 2015 with human codes for classification of emotion.

Type of emotion		Human codes					
		Affect	Positive	Negative	Anxiety	Anger	Sad
**LIWC^a^** **2001**							
	Affect	0.50^b^	0.38^b^	0.34^b^	0.23^b^	0.12^b^	0.17^b^
	Positive	−0.03^b^	0.47^b^	0.00	−0.01^b^	0.00	−0.01
	Negative	0.89^b^	0.00	0.58^b^	0.40^b^	0.20^b^	0.30^b^
	Anxiety	0.47^b^	0.01^b^	0.54^b^	0.74^b^	0.02^b^	−0.00
	Anger	0.33^b^	−0.00	0.23^b^	0.04^b^	0.49^b^	0.00
	Sad	0.42^b^	0.01	0.29^b^	0.00	0.00	0.52^b^
**LIWC 2007**							
	Affect	0.53^b^	0.35^b^	0.32^b^	0.21^b^	0.11^b^	0.16^b^
	Positive	−0.03^b^	0.43^b^	−0.00	−0.01^b^	−0.00	−0.01^b^
	Negative	1.00^c^	0.00	0.55^b^	0.38^b^	0.19^b^	0.28^b^
	Anxiety	0.56^b^	0.01^b^	0.50^b^	0.65^b^	0.02^b^	−0.00
	Anger	0.34^b^	−0.01	0.19	0.00	0.46^b^	0.00
	Sad	0.45^b^	0.01^c^	0.28^b^	0.00	0.00	0.50^b^
**LIWC 2015**							
	Affect	0.55^b^	0.36^b^	0.35^b^	0.21^b^	0.11^b^	0.15^b^
	Positive	−0.30	0.43^b^	−0.00	−0.01^b^	−0.00	0.01^b^
	Negative	1.00^b^	0.00	0.54^b^	0.37^b^	0.20^b^	0.27^b^
	Anxiety	0.55^b^	0.01^b^	0.49^b^	0.66^b^	0.02^b^	−0.00
	Anger	0.33^b^	−0.00	0.21^b^	−0.00	0.51^b^	0.00
	Sad	0.47^b^	0.01^c^	0.31^b^	0.00	0.00	0.57^b^

^a^LIWC: Linguistic Inquiry and Word Count.

^b^*P*<.001.

**Table 3:** Correlation matrix comparing Linguistic Inquiry and Word Count 2001, 2007, and 2015.

Type of emotion		2001						2007							2015						
		Affect	Positive	Negative	Anxiety	Anger	Sad		Affect	Positive	Negative	Anxiety	Anger	Sad		Affect	Positive	Negative	Anxiety	Anger	Sad
**2001**																					
	Affect	—^a^	—	—	—	—	—		—	—	—	—	—	—		—	—	—	—	—	—
	Positive	0.81^b^	—	—	—	—	—		—	—	—	—	—	—		—	—	—	—	—	—
	Negative	0.57^b^	−0.02^b^	—	—	—	—		—	—	—	—	—	—		—	—	—	—	—	—
	Anxiety	0.30^b^	−0.01^b^	0.53^b^	—	—	—		—	—	—	—	—	—		—	—	—	—	—	—
	Anger	0.21^b^	−0.01^b^	0.36^b^	0.05^b^	—	—		—	—	—	—	—	—		—	—	—	—	—	—
	Sad	0.26^b^	−0.01^b^	0.46^b^	0.03^b^	−0.00	—		—	—	—	—	—	—		—	—	—	—	—	—
**2007**																					
	Affect	0.86^b^	0.70^b^	0.50^b^	0.27^b^	0.18^b^	0.23^b^		—	—	—	—	—	—		—	—	—	—	—	—
	Positive	0.69^b^	0.86^b^	−0.03^b^	−0.01^b^	−0.01^b^	−0.01^b^		0.81^b^	—	—	—	—	—		—	—	—	—	—	—
	Negative	0.51^b^	−0.03^b^	0.91^b^	0.48^b^	0.33^b^	0.42^b^		0.55^b^	−0.03^b^	—	—	—	—		—	—	—	—	—	—
	Anxiety	0.31^b^	−0.01^b^	0.56^b^	0.85^b^	0.04^b^	0.02^b^		0.31^b^	−0.02^b^	0.57^b^	—	—	—		—	—	—	—	—	—
	Anger	0.19^b^	−0.01^b^	0.33^b^	−0.00	.086^b^	−0.00		0.20^b^	−0.01^b^	0.36^b^	−0.00	—	—		—	—	—	—	—	—
	Sad	0.26^b^	−0.01^b^	0.46^b^	0.02^b^	−0.00	0.93^b^		0.25^b^	−0.01^b^	0.45^b^	0.02^b^	−0.03	—		—	—	—	—	—	—
**2015**																					
	Affect	0.85^b^	0.68^b^	0.49^b^	0.26^b^	0.18^b^	0.23^b^		0.95^b^	0.77^b^	0.54^b^	0.31^b^	0.19^b^	0.25^b^		—	—	—	—	—	—
	Positive	0.67^b^	0.84^b^	−0.03^b^	−0.01^b^	−0.01^b^	−0.01^b^		0.77^b^	0.95^b^	−0.03^b^	−.02^b^	−0.01^b^	−0.01^b^		0.81^b^	—	—	—	—	—
	Negative	0.50^b^	−0.03^b^	0.89^b^	0.47^b^	0.33^b^	0.42^b^		0.53^b^	−0.03^b^	0.97^b^	0.56^b^	0.34^b^	0.45^b^		0.55^b^	−0.03^b^	—	—	—	—
	Anxiety	0.31^b^	−0.01^b^	0.54^b^	0.82^b^	0.05^b^	0.02^b^		0.31^b^	−0.02^b^	0.56^b^	0.97^b^	0.01^b^	0.02^b^		0.31^b^	−0.02^b^	0.55^b^	—	—	—
	Anger	0.18^b^	−.01^b^	0.32^b^	−0.00	0.80^b^	−0.00		0.18^b^	−0.01^b^	0.32^b^	−0.00	0.91^b^	−0.00		0.18^b^	−0.01^b^	0.33^b^	0.00	—	—
	Sad	0.28^b^	−0.01^b^	0.50^b^	0.02^b^	−0.00	0.88^b^		0.26^b^	−0.01^b^	0.47^b^	0.02	−0.00	0.90^b^		0.26^b^	−0.01^b^	0.47^b^	0.02^b^	−0.00	—

^a^Not applicable.

^b^*P*<.01.

### Relationship Between LIWC 2001 and LIWC 2007 Coding Methods

#### Sensitivity

Sensitivity captured the proportion of total emotion words identified by human raters as being representative of emotional expression that were also captured by LIWC 2001, LIWC 2007, or LIWC 2015. Sensitivity for overall emotional expression was good (eg, greater than 0.80, equivalent to at least 80% accuracy) for all 3 versions: LIWC 2001 (0.858), LIWC 2007 (0.896), and LIWC 2015 (0.904; Table 4). Additionally, all 3 versions demonstrated good sensitivity for positive emotion, negative emotion, and anxiety, with results ranging from 0.819 to 0.892. Alternatively, LIWC 2001 and LIWC 2007 demonstrated poor (less than 0.80) performance in anger (0.663 and 0.679, respectively) and sadness (0.699 and 0.718, respectively), whereas LIWC 2015 demonstrated good performance for sadness (0.856) and poor performance for anger (0.695). Compared with LIWC 2001, LIWC 2007 and LIWC 2015 produced significantly higher identification of emotional expression in the categories of overall emotion and positive emotion. Additionally, LIWC 2015 demonstrated higher sensitivity for the category of sadness when compared with LIWC 2001 and LIWC 2007. Notably, there were no differences between LIWC 2001, LIWC 2007, and LIWC 2015 for negative emotions, anxiety, or anger.

**Table 4:** Linguistic Inquiry and Word Count 2001, 2007, and 2015 sensitivity and specificity with 95% CI (N=63)^a^.

Emotion categories	2001 sensitivity (95% CI)	2007 sensitivity (95% CI)	2015 sensitivity (95% CI)	2001 specificity (95% CI)	2007 specificity (95% CI)	2015 specificity (95% CI)
Total affect	0.858^a,b^ (0.845-0.871)	0.896^b^ (0.884-0.906)	0.904^c^ (0.894-0.915)	0.967 (0.966-0.968)	0.955 (0.954-0.956)	0.955 (0.954-0.956)
Positive emotion	0.873^b,c^ (0.855-0.889)	0.913^b^ (0.898-0.927)	0.928 (0.915-0.941)	0.976 (0.975-0.977)	0.967 (0.966-0.967)	0.967 (0.958-0.976)
Negative emotion	0.822 (0.803-0.839)	0.814 (0.793-0.834)	0.810 (0.789-0.830)	0.990 (0.990-0.991)	0.987 (0.987-0.988)	0.988 (0.982-0.994)
Anxiety	0.862 (0.829-0.888)	0.892 (0.863-0.916)	0.883 (0.856-0.910)	0.998 (0.998-0.999)	0.997 (0.996-0.997)	0.997 (0.993-1.00)
Anger	0.663 (0.591-0.729)	0.679 (0.607-0.744)	0.695 (0.629-0.761)	0.998 (0.998-0.999)	0.998 (0.998-0.999)	0.999 (0.995-1.00)
Sadness	0.699^c^ (0.645-0.748)	0.718^d^ (0.664-0.766)	0.856^c,d^ (0.818-0.895)	0.997 (0.997-0.998)	0.997 (0.997-0.997)	0.997 (0.991-1.00)

^a^*P* value corrected after Bonferroni *P*<.0021 (*P*=alpha/N).

^b^Significant difference between Linguistic Inquiry and Word Count 2001 and Linguistic Inquiry and Word Count 2007 at *P*<.0021.

^c^Significant difference between Linguistic Inquiry and Word Count 2001 and Linguistic Inquiry and Word Count 2015 at *P*<.0021.

^d^Significant difference between Linguistic Inquiry and Word Count 2007 and Linguistic Inquiry and Word Count 2015 at *P*<.0021.

#### Specificity

Specificity measured the proportion of nonemotional words that were accurately coded by LIWC 2001, LIWC 2007, or LIWC 2015 as not being indicative of emotion. Specificity for LIWC 2001, LIWC 2007, and LIWC 2015 was exceptional in all emotion categories (Table 4). Specificity values for LIWC 2001, LIWC 2007, and LIWC 2015 ranged from 0.955 for total emotional expression to 0.999 for anger. There were no differences in overall emotional expression, positive emotions, negative emotions, anxiety, anger, or sadness between LIWC 2001 and LIWC 2007.

#### PPV

PPV measured the probability that a word identified by LIWC 2001, LIWC 2007, and LIWC 2015 as being representative of emotional expression was in agreement with human rater coding of emotional expression. PPV for LIWC 2001, LIWC 2007, and LIWC 2015 was poor in all emotion categories (Table 5). The PPV values for all 3 versions ranged from 0.207 to 0.640. LIWC 2001’s PPV was significantly better than LIWC 2007’s and LIWC 2015’s PPV in total emotion, negative emotion, and anxiety and was significantly better than LIWC 2007’s PPV in positive emotion. Notably, there were no differences in anger or sadness.

**Table 5:** Linguistic Inquiry and Word Count 2001, 2007, and 2015 positive predictive value and negative predictive value with 95% CI (N=63)^a^.

Emotion categories	2001 PPV^b^ (95% CI)	2007 PPV (95% CI)	2015 PPV (95% CI)	2001 NPV^c^ (95% CI)	2007 NPV (95% CI)	2015 NPV (95% CI)
Total affect	0.326^d,e^ (0.315-0.336)	0.268^d^ (0.259-0.277)	0.270^e^ (0.254-0.286)	0.997 (0.997-0.997)	0.997 (0.997-0.998)	0.998 (0.996-0.999)
Positive Emotion	0.256^d^ (0.244-0.268)	0.207^d^ (0.197-0.217)	0.211 (0.191-0.231)	0.998 (0.998-0.998)	0.999 (0.999-0.999)	0.999 (0.997-1.00)
Negative Emotion	0.498^d,e^ (0.479-0.516)	0.377^d^ (0.361-0.395)	0.373^e^ (0.348-0.398)	0.998 (0.998-0.998)	0.998 (0.998-0.998)	0.998 (0.996-1.00)
Anxiety	0.640^d^ (0.605-0.675)	0.477^d^ (0.446-0.508)	0.496 (0.454-0.538)	0.999 (0.999-0.999)	0.999 (0.999-0.999)	0.999 (0.996-1.00)
Anger	0.357^e^ (0.307-0.409)	0.317 (0.273-0.366)	0.375^e^ (0.306-0.444)	0.999 (0.999-0.999)	0.999 (0.999-0.999)	0.999 (0.995-1.00)
Sadness	0.389 (0.349-0.431)	0.351 (0.315-0.389)	0.377 (0.324-0.430)	0.999 (0.999-0.999)	0.999 (0.999-0.999)	0.999 (0.996-1.00)

^a^*P* value corrected after Bonferroni *P*<.0021 (*P*=alpha/N).

^b^PPV: positive predictive value.

^c^NPV: negative predictive value.

^d^Significant difference between Linguistic Inquiry and Word Count 2001 and Linguistic Inquiry and Word Count 2007 at *P*<.0021.

^e^Significant difference between Linguistic Inquiry and Word Count 2001 and Linguistic Inquiry and Word Count 2015 at *P*<.0021.

#### NPV

NPVs measured the probability that a word not identified as emotion by LIWC 2001, LIWC 2007, and LIWC 2015 agreed with raters’ judgment that the word was not associated with emotional expression. LIWC 2001, LIWC 2007, and LIWC 2015 have excellent NPVs across all emotion categories (Table 5). The NPV for LIWC 2001, LIWC 2007, and LIWC 2015 ranged from 0.997 for total emotional expression to 0.999 for anxiety, anger, and sadness. There were no significant differences between LIWC 2001, LIWC 2007, and LIWC 2015 with regard to NPV.

#### F Score

The results of the *F* score were compared using a test of difference, which was conducted to determine whether the difference between two proportions was significant, and it revealed that LIWC 2001 was significantly more precise in its evaluation of total emotional expression, positive emotion, and anxiety in comparison with LIWC 2007. Additionally, LIWC 2001 showed significantly more precision in its evaluation of total affect, positive emotion, negative emotion, and anxiety in comparison with LIWC 2015. Notably, there were no significant differences in accuracy between LIWC 2007 and LIWC 2015 (Table 6).

**Table 6:** Linguistic Inquiry and Word Count 2001, 2007, and 2015 F score values.

Emotion categories	2001 *F* score	2007 *F* score	2015 *F* score	Difference 2001-2007	Difference 2001-2015	Difference 2007-2015
Total affect	0.472	0.413	0.415	<.0001^a^	<.0001^a^	.875
Positive emotion	0.396	0.337	0.344	.0007^a^	.003^a^	.683
Negative emotion	0.561	0.516	0.511	.014	.007^a^	.786
Anxiety	0.735	0.622	0.635	<.0001^a^	.0003^a^	.654
Anger	0.464	0.433	0.487	.542	.654	.291
Sadness	0.499	0.497	0.523	.497	.544	.198

^a^*P* value corrected after Bonferroni *P*<.0083 (*P*=alpha/N).

## Discussion

Successive versions of LIWC have become increasingly sensitive to identifying emotional expression. Our hypothesis that LIWC 2015 would significantly differ from LIWC 2001 and 2007 and be more efficacious in emotional identification was not supported. LIWC 2007 and 2015 were able to increase the previously established strength of LIWC 2001 in the identification of overall emotional expression emotion and positive emotion, whereas LIWC 2015 also increases the strength in the identification of sadness. However, LIWC 2007 and 2015 both exacerbate the existing weakness of LIWC 2001 in that many of the words it identifies as emotion do not match the ratings of human coders. Regarding the identification of nonemotional words, there was no improvement by LIWC 2015 or LIWC 2007. These findings suggest that although LIWC 2015 and LIWC 2007 had higher levels of emotional identification, more words were also inaccurately classified as emotion, compared with LIWC 2001. Therefore, although LIWC 2001, LIWC 2007, and LIWC 2015 measure a number of domains other than emotional expression, our findings suggest that all 3 versions, LIWC 2001, LIWC 2007, and LIWC 2015, have excellent sensitivity for detecting emotional expression, but LIWC 2001 has more precision with respect to PPV—the words it identifies as representing emotion are more likely than LIWC 2007 and LIWC 2015 to be in agreement with human raters.

The sensitivity levels for all 3 versions of LIWC indicate strength with regard to the identification of emotional content, such that they were highly sensitive to the identification of emotional expression. This is exceptionally important when analyzing text data where the expected prevalence of emotional expression is low or where the risk of overidentification is much lower than the risk of under-identification (eg, in evaluating suicide risk), where a missed instance could have grave consequences. There are many applications for which sensitivity, rather than accuracy, may be preferable, and LIWC 2007 and LIWC 2015 demonstrate an improved performance in sensitivity relative to LIWC 2001. However, there are potential consequences to the overidentification of emotional expression, particularly when accurate emotion recognition is required for a specific utterance.

In contrast to sensitivity, the PPV was fairly poor for LIWC 2001, LIWC 2007, and LIWC 2015. LIWC 2001 produced a significantly more precise performance with regard to PPV compared with LIWC 2007 and LIWC 2015, whereas there were no significant differences between LIWC 2007 and LIWC 2015. Evaluation of the F score, which balances both PPV and sensitivity, revealed that LIWC 2001 was superior to LIWC 2007 in the emotional identification of overall affect, positive emotions, and anxiety. The remaining categories were not significantly different, indicating that LIWC 2001 and LIWC 2007 performed similarly in their identification of those emotion categories (eg, negative emotion, anger, and sadness). Additionally, with regard to *F* scores, LIWC 2001 was superior to LIWC 2015 in terms of total overall affect, positive emotions, negative emotions, and anxiety. The remaining categories were not significantly different, indicating that LIWC 2001 and LIWC 2015 performed similarly in their identification of those emotion categories (eg, anger and sadness). Finally, there were no significant differences in the F scores of emotional identification between LIWC 2007 and LIWC 2015 across the emotion categories (eg, overall affect, positive emotions, negative emotions, anxiety, anger, and sadness). These results indicate that LIWC 2001 is more inclined to accurately identify emotion in accordance with human coders when compared with LIWC 2007 and LIWC 2015, wherein one balances the problem of overidentification against the problem of under-identification, which is pertinent when considering at-risk populations (eg, suicidal patients). Considering that human coders are the gold standard for emotional identification and that LIWC 2001 provides results that are most similar to those of human coders, LIWC 2001 is superior to LIWC 2007 and LIWC 2015.

As previously mentioned, Pennebaker et al [14] made a number of changes to the LIWC 2007 and LIWC 2015 dictionaries, which may have resulted in the decrease in precision of the subsequent versions. Although the alterations to LIWC 2007 and LIWC 2015 resulted in improvements in sensitivity, these changes did not improve LIWC 2001’s previously established flaws. The alterations to the LIWC 2007 and LIWC 2015 dictionary may have resulted in the increased identification but a decrease in the precision of the identification.

LIWC 2001 may present as superior in its emotional identification over LIWC 2007 and LIWC 2015, yet the accuracy in its performance is highly dependent upon the population being evaluated. The PPV is dependent upon the prevalence in the population, meaning it can vary based on the sample utilized, whereas sensitivity may remain the same despite what population is being evaluated [29]. More specifically, cancer patients have been found to express more emotion than a physically healthy population [30], meaning that the prevalence of expressed emotion is higher for the sample utilized in this study than that of the general population. Considering prevalence rates or emotional expression in the cancer population, LIWC 2001 and LIWC 2007 are likely to produce poorer PPVs if being utilized with the emotional expression of a nonclinical population. Text that is likely to have nuanced emotion, such as is often the case in the experience of cancer, will also be less likely to be related to accuracy when coded by LIWC. LIWC 2001 and LIWC 2007 currently have a high rate of false positives, which may increase when evaluating a less emotionally expressive population or decrease when evaluating a more emotionally expressive population. Ultimately, the LIWC programs would benefit from further validation utilizing alternative populations with varying levels of emotional expression.

The initial validation of LIWC 2001, 2007, and 2015 relied heavily on simple correlations between LIWC codes and judge’s ratings of emotional content. Correlation analyses describe the extent to which the variables covary, but not the accuracy of identification. For this, measures of testing accuracy are more appropriate. Conducting analyses such as a test of proportions allows users to see the weaknesses and strengths of the relationship and what factors contribute to the strengths of that relationship. Additionally, future studies may also benefit from evaluating writers’ intentions related to emotional expression to further validate both LIWC and human coders’ ratings. For example, the LIWC classification results (eg, negative emotion) could have been reviewed while also conducting a review with the text writers, ensuring that they intended to express negative emotions, thus confirming the LIWC classification. A machine learning approach, in which rules for coding are created as the raw data are under analysis, in combination with LIWC is another possibility for increasing the overall accuracy as well as making the coding procedure more sample dependent. With a machine learning approach, automated coding rules or algorithms are generated from patterns seen in other coding methods, such as manual coding, potentially allowing for increased accuracy without the time-consuming nature of manual coding [13]. Emotions are multifaceted, making them much more difficult to accurately identify when simplified to one modality. It is important to note that there are many other ways to code emotion in text, and this study focused on validating a program that is commonly used in the field of web-based behavioral sciences.

It must be noted that there are some limitations to this study. The narratives utilized in this study were obtained from women diagnosed with breast cancer. Research has indicated that female cancer patients express more emotion than male counterparts [30]. Additionally, cancer patients are more inclined to endorse affective disorders, such as anxiety, which may impact their emotional expression [31]. Additionally, based on the specific circumstances these women faced (eg, cancer diagnosis, treatment, and outcomes), this could have limited the range of emotions that may have been discussed compared with a healthy population. On the basis of the population utilized, results may be limited to cancer survivors rather than the general population. Furthermore, there were few emotions evaluated (eg, overall affect, positive emotions, negative emotions, anger, anxiety, and sadness), which does not reflect the full range of emotions experienced. This limited range of emotions measured may not accurately reflect the emotions expressed (eg, frustration, excitement, and fear). Finally, the sample utilized was highly homogenous with respect to gender, ethnicity, level of education, and even health status, further limiting the generalizability of the findings.

In contrast to the sample, additional limitations present within this study include intricacies specific to LIWC. As previously mentioned, the classification of the word “like” has been altered across each iteration of LIWC. Most notably, when utilizing LIWC 2015 in text analysis, it may be most accurate to manually evaluate each instance of the word “like” to determine the contextual meaning of the word to ensure appropriate classification of each word. Despite this, manual evaluation determining the context of the word “like” was not performed before conducting automatic analysis with LIWC 2015, as this defeats the utility and efficiency that automatic programs of text analysis offer.

Although human coders are the gold standard for emotional identification in text data, due to the time and cost associated with evaluating such large volumes of data, human coding is not always practical. In addition, manual coding of text, although considered the gold standard, is not entirely accurate. There is inevitably some ambiguity in any attempt to capture the emotion expressed by another person. Text-based data also leave absent, nonverbal expression, leaving fewer cues to code. On the basis of the results, LIWC 2001 is clearly superior to LIWC 2007 and LIWC 2015 with respect to overall precision, but LIWC 2007 and LIWC 2015 are more sensitive to identifying overall expressed emotions. The PPV is highly dependent on the prevalence of emotion in the specific population, such that the more emotion presented in a population, the more accurate the analysis will likely be. Considering the high prevalence of emotion in a cancer population and that LIWC 2001 performed significantly better than LIWC 2007 and LIWC 2015, this indicates that for a population with much less emotional expression, LIWC 2007 and LIWC 2015 will still perform significantly poorer than LIWC 2001. LIWC 2001 seems to have good validity in emotional identification and presents as a reasonable tool for identification of emotion in text data, which is important in the increasingly digital world.

## Abbreviations

LIWC: Linguistic Inquiry and Word Count
NPV: negative predictive value
PPV: positive predictive value
